# Big Data, Decision Models, and Public Health

**DOI:** 10.3390/ijerph19148543

**Published:** 2022-07-13

**Authors:** Chien-Lung Chan, Chi-Chang Chang

**Affiliations:** 1 Department of Information Management, Yuan Ze University, Taoyuan 32003, Taiwan; clchan@saturn.yzu.edu.tw; 2 Innovation Center for Big Data and Digital Convergence, Yuan Ze University, Taoyuan 32003, Taiwan; 3 School of Medical Informatics, Chung Shan Medical University & IT Office, Chung Shan Medical University Hospital, Taichung 40201, Taiwan; 4 Department of Information Management, Ming Chuan University, Taoyuan 33300, Taiwan

## 1. Introduction

As the digital era unfolds, the volume and velocity of environmental, population, and public health data are rapidly increasing. In recent decades, big data analytic techniques such as statistical analysis, data mining, machine learning, and deep learning have made significant progress and have attracted the attention of researchers and scientists in a variety of applications [1]. Making decisions based on concrete evidence is particularly important and has a substantial impact on public health and program implementation. In addition to working with human health and medical information, we must also consider scientific integrity issues such as privacy, data sharing, bias, and statistical inference. For these reasons, this Special Issue focuses on the use of big data analytics and forms of public health decision making that utilize the decision model, spanning from theory to practice.

The Special Issue received 64 high-quality submissions. The manuscripts were carefully blind peer-reviewed by at least two referees, according to the *International Journal of Environmental Research and Public Health* (*IJERPH*) review procedure, and 25 papers were selected for publication in this Special Issue. We have successfully addressed the critical research needs for big data, decision models, and public health in this Special Issue. In addition, the guest editors thank the *International Journal of Environmental Research and Public Health* for this wonderful opportunity to organize this Special Issue. We also thank the contact editor, Sherry Cao, for her patience and support.

## 2. The Organization of This Special Issue

With the previous Special Issue on ’Big Data, Decision Models, and Public Health’ [1] being successful, similar to the first edition, this second Special Issue covers five important themes. The first theme looks at preventive medicine and risk assessment. The second theme looks at the forecasting models to support healthcare policies. The third theme considers big data analytics to improve public health and chronic diseases. The fourth theme investigates the potential risks and benefits associated with disease control. Finally, the fifth theme describes the health-related quality of life.

### 2.1. Preventive Medicine and Risk Assessment

The most important aspect of healthcare activities is to provide preventive care [2,3,4,5]. The first paper, “*Risk Assessment of Early Lung Cancer with LDCT and Health Examinations*”, by Chang et al. [6], examines the risk of stage I lung cancer in the general population, not limited to smokers, in a retrospective study conducted in Taiwan. The results confirm that older age; higher white blood cell count (WBC); higher blood urea nitrogen (BUN); diabetes; gout; chronic obstructive pulmonary disease (COPD); other cancers; and the presence of spiculation, ground glass opacity (GGO), and part solid nodules are associated with a higher risk of lung cancer. Subjects with calcification; solid nodules; nodules in the middle lobes; more nodules; and diseases related to the thyroid, liver, and digestive systems were at lower risk.

The challenge for decision makers and occupational medical professionals is the incorporation of this information into daily practices and health promotion campaigns. The second paper, “*Factors Associated with Cardiovascular Disease Risk among Employees at a Portuguese Higher Education Institution*”, by Brandão et al. [7], estimated the prevalence of risk factors for cardiovascular disease (CVD) and assessed the CVD risk in Portugal. According to the results, hypercholesterolemia (43.2%) was the most common CVD risk factor. Furthermore, the consumption of soft drinks twice or more per week significantly increased the risk of CVD, as did the lack of regular exercise and the lack of daily fruit consumption. This preliminary study offers new information to the medical community and decision makers on risk factors associated with CVD in the population.

Health education interventions can have positive effects in terms of change in behavior and literacy. Mobile health services are a supportive technique that has gradually been introduced to support patients in their self-management of chronic disorders. The third paper, “*Effects of Mobile Application Program (App)-Assisted Health Education on Preventive Behaviors and Cancer Literacy among Women with Cervical Intraepithelial Neoplasia*”, by Lee et al. [8], enrolled women with cervical intraepithelial neoplasia (CIN); it aimed to examine the effects of health education on preventive behaviors and cancer literacy and to compare the effects of health education with those of traditional book form health education. The self-efficacy of behavior, especially the concept of risk control, was investigated and improved due to educational interventions.

Prostate cancer is the second most frequently diagnosed cancer worldwide and is among the most common causes of cancer mortality among men. Risk factors such as ethnicity, family history, diet, smoking, and somatic genomic alterations have been suggested to be associated with prostate cancer carcinogenesis. In the fourth paper, “*Impact of Matrix Metalloproteinase-11 Gene Polymorphisms on Biochemical Recurrence and Clinicopathological Characteristics of Prostate Cancer*”, by Hsieh et al. [9], the results demonstrate that the MMP-11 polymorphisms, particularly rs131451, were associated with late-stage tumor development in prostate cancer with biochemical recurrence. The MMP-11 SNP rs131451 may contribute to tumor development in prostate cancer patients with biochemical recurrence.

### 2.2. Forecasting Models to Support Healthcare Policies

The health services and support to decision makers in implementing targeted health policies are examined in this theme [10,11,12,13,14]. The complex dynamics of human mobility and the variable intensity of local outbreaks make it difficult to measure the factors of epidemic transmission. The first paper, “*Suburban Road Networks to Explore COVID-19 Vulnerability and Severity*”, by Uddin et al. [15], found that the degree-of-centrality measure for suburban road networks was a significant predictor of both vulnerability and severity for COVID-19. The findings could provide stakeholders and policymakers with practical insights into developing timely strategies and policies to prevent infectious pandemics.

Explainability is one of the key characteristics of healthcare support systems. The second paper, “*Deep Ensemble Learning Approaches in Healthcare to Enhance the Prediction and Diagnosing Performance: The Workflows, Deployments, and Surveys on the Statistical, Image-Based, and Sequential Datasets*”, by Nguyen et al. [16], proposed three deep ensemble learning (DEL) approaches, each with stable and reliable performances, that are workable for the above-mentioned data types. The results of the experiment showed that our proposed approaches achieve better performance than traditional machine learning and deep learning techniques on sequential, image-based, and sequential benchmark datasets.

Numerous scientific studies have shown that universal single-payer health insurance does not necessarily promote the balanced development of primary care. The third paper, “*Exploration of Preventable Hospitalizations for Colorectal Cancer with the National Cancer Control Program in Taiwan*”, by Hung et al. [17], conducted a cohort study to examine long-term trends in preventable hospitalizations (PH) and to reduce the incidence and severity of cancer. Differences in PH rates between rural and urban areas can also be used as a reference to achieve an equitable distribution of medical resources.

The use of predictive analytics provides a “filter” for first-level identification that was traditionally performed by clinicians. The fourth paper, “*Implementing an Individual-Centric Discharge Process across Singapore Public Hospitals*”, by Ng and Tan [18], developed models using machine learning algorithms including gradient boosting machine, random forest, lasso, and logistic regression. As a result of its near-real-time capabilities, this study demonstrated the viability of using artificial intelligence (AI) to build a near-real-time nationwide individual-centric discharge prediction tool, as well as the critical factors for successful implementation.

Land transport accidents are a major public health issue around the world. There is limited population-based epidemiological evidence of the risk of land transport accidents after peripheral vestibular disorders (PVD). In the fifth paper, “*A Nationwide Population-Based Study on the Association between Land Transport Accident and Peripheral Vestibular Disorders*”, by Lin et al. [19], the results show that subjects with prior diagnosis of peripheral vestibular disorders have over twofold higher odds of land transport accidents than the controls in Taiwan. It may help physicians advise vestibular impaired patients about possible safety threats regarding driving a motor vehicle and provide guidance for future legislators and enforcement laws.

The most critical goal of subarachnoid hemorrhage (SAH) management is the prevention of rebleeding by early repair of the unsecured aneurysm through surgical clipping or endovascular coiling. However, there is no answer to the financial cost of the two techniques for aneurysmal SAH. In the fifth paper, “*Long-Term Medical Resource Consumption between Surgical Clipping and Endovascular Coiling for Aneurysmal Subarachnoid Hemorrhage: A Propensity Score–Matched, Nationwide, Population-Based Cohort Study*”, by Lo et al. [20], the results revealed a shorter cumulative hospital stay and ICU stay in the coiling group for aneurysmal SAH and less possible subsequent surgical complications and recurrence. Furthermore, the total benefits of a lower consumption of medical resources in patients with aneurysmal SAH receiving coiling compared to those receiving clipping can be used to establish further health policies. The results of the study can provide policymakers with sufficient information to reconsider the entire reimbursement scheme from a holistic perspective.

### 2.3. Big Data Analytics to Improve Public Health and Chronic Diseases

A major focus of this theme is on how big data analytics can improve public health and chronic diseases [21,22,23,24,25,26]. The first paper, “*Study on the Impact of Income Gap on the Health Level of Rural Residents in China*”, by Guo et al. [27], examined the heterogeneity of the impact of income gap on the health level of different rural households. The results indicate that households with low social capital, those without rental income, and people without elderly household heads are the most affected by the formation of income gaps. In addition to improving the tax system, providing tax relief policies to some people with economic difficulties, and improving the efficiency of government funds, the government should make further improvements to the redistribution system. In addition to balancing the income level for rural residents, it is important to address the distribution gap.

Neurodegenerative disorders such as Alzheimer’s disease (AD) have an insidious onset and are irreversible in nature. Patients with mild cognitive impairment (MCI) are very likely to develop Alzheimer’s disease. Therefore, a timely diagnosis of unstable patients with MCI is crucial to slowing the progression of AD in these patients. The second paper, “*A Machine Learning Classifier to Predict Stable MCI Patients Using Gene Biomarkers*”, by Lin et al. [28], developed feature selection and machine learning algorithms to predict stable MCI patients based on gene biomarkers from blood samples. A total of 29 genes (31 probes) were analyzed from the Alzheimer’s Disease Neuroimaging Initiative (ADNI) database to determine if they were biomarkers capable of predicting stable patients with MCI based on two datasets. A precision medicine prediction model could help identify stable patients with MCI and provide medical doctors and patients with new first-tier diagnosis options.

Due to the heterogeneity of CKD progression, it is critical to develop a longitudinal diagnosis and prognosis for patients with CKD. In the third article, “*Development of a Longitudinal Diagnosis and Prognosis in Patients with Chronic Kidney Disease: Intelligent Clinical Decision-Making Scheme*”, by Shih et al. [29], the proposed auto-ML scheme can automatically select the level of each strategy to associate with a classifier that consists of four main parts: classification pipeline, cross-validation, Taguchi method, and improved strategies. The experimental results showed that age, creatinine, high blood pressure, and smoking are important risk factors, which has been proven in previous studies. The auto-ML scheme shed light on the possibility of evaluation for the effectiveness of one or a combination of those risk factors. This methodology may provide essential information and longitudinal change for personalized treatment in the future.

It is likely to underestimate the risk in epidemiological studies when the study population is relatively healthier than the comparison population. The fourth paper, “*Application of Standardized Proportional Mortality Ratio to the Assessment of Health Risk in Relatively Healthy Populations: Using a Study of Cancer Risk in Telecommunication Workers with Excess Exposure to Acid Mists as an Example*”, by Ker et al. [30], used a case study on the cancer risk of workers exposed to acid mists, a well-documented carcinogen, to demonstrate that using proportional mortality ratios (PMRs) is more appropriate than using mortality ratios to assess risk in terms of mortality. The results showed that PMR can detect increases in mortality when a study population is generally healthier than the comparison population and called for further studies on the possible carcinogenic effects of low-level acid mist exposures in the stomach.

Unlike traditional statistics, the multiple criteria decision-making (MCDM) approach can support decision-makers in making rational judgments by considering multiple factors simultaneously, without presumed probabilistic distributions. The fifth paper, “*Clinical Knowledge Supported Acute Kidney Injury (AKI) Risk Assessment Model for Elderly Patients*”, by Shen et al. [31], uses acute kidney injury (AKI) as an empirical case, based on the clinical experience of doctors, and develops a flexible knowledge-based system to support clinical prognoses. Statistics are still the cornerstone of understanding many illnesses in clinical research. A well-designed meta-analysis may explore and examine inconclusive disease patterns from credible sources, with rigorous control and testing. Contributions can be seen in its work.

Despite the impacts of parapharyngeal and retropharyngeal abscesses (PRPAs) and the high cost burden, there are few long-term population-based epidemiological data on the incidence and mortality of PRPAs. The sixth paper, “*A Nationwide Population-Based Study on the Incidence of Parapharyngeal and Retropharyngeal Abscess—A 10-Year Study*”, by Yang et al. [32], aimed to estimate the incidence of PRPA among the Taiwan population, including age and gender distributions, as well as longitudinal trends, using insurance claims data in Taiwan’s National Health Insurance databases. The higher incidence rate in this study compared to other countries emphasizes the importance of early detection, especially for healthcare workers. Furthermore, it may warrant further research on the impact of healthcare systems.

### 2.4. The Disease Control and Treatment Outcome

This theme investigates the potential risks and benefits associated with disease control [33,34,35,36,37]. Data mining is a technique that allows for the analysis of large data sets, enabling the identification of previously unknown relationships and new structures. Data extraction can provide valuable information for creating national or global strategies and at all stages of decision-making. The first paper, “*Challenges and Drawbacks of the EU Medical System Generated by the COVID-19 Pandemic in the Field of Health Systems’ Digitalization*”, by Țăran et al. [38], examines the impact of digitalization on several dimensions of health, with specific implications for their impact on the Coronavirus disease 2019 (COVID-19) pandemic in mind. The results of the study emphasize the need for effective national responses, as well as recommendations, priorities, and objectives to strengthen health systems at the European level. Additionally, the results point to the need for a new approach to policy, governance, investment, health spending, and the provision of digital services.

It is important to consider multiple strategies when dealing with a pandemic such as COVID-19. The continuous relationship over time among the variables involved renders an approach with Bayesian networks and Structural Equations Models particularly suitable. The second paper, “*Integrated Analysis of Behavioural and Health COVID-19 Data Combining Bayesian Networks and Structural Equation Models*”, by Kenett et al. [39], aims to examine the effects of pandemic management and mitigation policies on pandemic spread and population activity. By modeling data from health registries and Google mobility data, decision makers can conduct scenario analyzes to help design adequate pandemic management policies. To effectively control pandemics, social, public health, behavioral, and economic factors, as well as an assessment of the population’s ability of the population must be considered to maintain restrictions over time.

Infections of the urinary tract and upper respiratory tract are the most common during pregnancy. The safety and efficacy of antibiotic therapy (AT) during pregnancy are limited, with even less information available according to the trimester of its use. The third paper, “*Use of Antibiotic Treatment in Pregnancy and the Risk of Several Neonatal Outcomes: A Population-Based Study*”, by Cantarutti et al. [40], aimed to evaluate the association between exposure to AT during pregnancy and short-term neonatal outcomes. They found that the risk of preterm birth, low birth weight, and low Apgar score was increased by 17, 11, and 16% in infants born to mothers with early exposure. Those exposed in the late pregnancy had an increased risk of preterm birth, low birth weight, and low Apgar scores of 25, 11, and 13%, respectively. A woman prescribed AT during pregnancy should be closely monitored to reduce the risk of neonatal outcomes.

Patient-centered care has become a central value in healthcare systems, and this represents a change in the traditional roles of patients and their families, from taking orders to becoming team members. Consequently, the trend in medical decision making has changed from passive informed consent to shared decision making (SDM). The fourth paper, “*Incorporating Patient Preferences into a Decision-Making Model of Hand Trauma Reconstruction*”, by Chang et al. [41], conducted a conjoint analysis (CA) to determine which factors are most important for patients who will undergo hand soft tissue reconstruction. Furthermore, both patients and physicians can use this information to determine what patients are willing to sacrifice to achieve their treatment goals. Understanding patient preferences for treatment options and considering which treatment factors are valued by patients may help guide decision making toward patient-centered care.

Several studies have reported an increase in autoimmune diseases worldwide. Myasthenia gravis (MG) is the most common disorder of the neuromuscular junction among autoimmune diseases. Despite this, the causes of Myasthenia gravis are not well understood. The fifth article, “*Absence of association between Previous Mycoplasma pneumoniae Infection and Subsequent Myasthenia Gravis: A Nationwide Population-Based Matched Cohort Study*”, by Chen et al. [42], investigated the relationship between mycoplasma pneumoniae infection and the subsequent development of MG. The study did not find a statistically significant association between mycoplasma pneumoniae infection and subsequent development of MG. More studies with a larger population and more cases of MG are needed to confirm this conclusion.

Risk factors are increasingly commonly used to predict risk in precision medicine. All patients undergoing lung resection surgery visit the preanesthetic consulting clinic for risk assessments, explanations, and discussions with anesthesiologists before general anesthesia. One of the most important topics in preanesthetic consultation clinics is the possibility of transferring to the ICU for staged weaning. The sixth paper, “*A Real-Time Artificial Intelligence-Assisted System to Predict Weaning from Ventilator Immediately after Lung Resection Surgery*”, by Chang et al. [43], determined the accuracy of pre-anesthetic evaluations, and a computerized artificial intelligence (AI) prediction model was developed based on critical, successful factors. Digitalization helps patients understand the risks of the inability to wean immediately, and the need for high-concentration oxygen and intensive care. Furthermore, during the pre-anesthetic consultation, both junior and senior anesthesiologists reported that the AI predictive system improved their ability to explain and communicate to patients and family members.

### 2.5. Health-Related Quality of Life

This theme describes the health-related quality of life [44,45,46]. The quality of the data plays a major role in the performance of recognition. The reason for an infant’s crying is challenging, since babies cannot communicate verbally to express their desires or needs. Indeed, this makes it difficult for parents to identify their infant’s health needs. The first paper, “*Deep Learning for Infant Cry Recognition*”, by Liang et al. [47], used the convolutional neural network (CNN) and long short-term memory (LSTM) to recognize infants’ necessities. As a result, all of the proposed methods were able to distinguish the cry of an infant in good health with high accuracy, precision, and recall. It may be possible to improve the robustness of the model by integrating video signals in the future.

A sentiment is a way of expressing an emotion. More attention should be placed on human-centered concepts in urban planning and design as the built environment and people become increasingly important. People’s sentiment and well-being are affected by the degree to which the physical environment meets their physical and psychological needs. The second paper, “*Exploring the Relationship between Urban Youth Sentiment and the Built Environment Using Machine Learning and Weibo Comments*”, by Duan et al. [48], based on data from Sina Weibo comments posted in Shanghai and using the machine learning algorithm, calculated the sentiment label and sentiment intensity of each comment. In addition, there were ten elements in five aspects selected to assess the built environment at different scales and to explore the correlations between built environment elements and sentiment intensity at different scales. This study found that urban youth have a higher proportion of both happy and sad sentiments, within which sad sentiments are more closely related to the built environment, and confirmed that the built environment has a great impact on sentiment.

Although previous research has shown the positive effects of social support or empathy on people’s mental health state and user contributions, few studies have examined the effects from the perspective of online mental health communities, which is a crucial source of online mental health support. The third article, “*Exploring the Effect of Social Support and Empathy on User Engagement in Online Mental Health Communities*”, by Chen et al. [49], focused on 22 mental health-related subreddits, and matched and compared users (1) who received social support with those who did not receive social support and users (2) who received more empathic social support with those who received less empathic social support. This study proves that social support and empathy are “contagious”. The results indicate the potential chain reaction of social support and empathy in online mental health communities. Furthermore, this study provides information on how online mental health communities can serve as a better place for people to spread social support.
